# Brief Evaluation of Olink Reveal Proximity Extension Assay for High-Throughput Proteomics: A Case Study Using NIST SRM 1950 and Two Spike-In Protein Standards

**DOI:** 10.1021/acs.jproteome.5c00571

**Published:** 2025-09-09

**Authors:** Yuri E. M. van der Burgt, Emile de Meijer, Magnus Palmblad

**Affiliations:** † Center for Proteomics and Metabolomics, 4501 Leiden University Medical Center, Postbus 9600, 2300 RC Leiden, The Netherlands; ‡ Department of Human Genetics, 4501 Leiden University Medical Center, Postbus 9600, 2300 RC Leiden, The Netherlands

**Keywords:** Olink Reveal, affinity proteomics, plasma

## Abstract

Plasma proteomics has regained attention in recent years through advancements in mass spectrometry instrumentation and sample preparation as well as new high-throughput affinity-based technologies. Here, we evaluate the analytical performance of the new Olink Reveal platform, a proximity extension assay (PEA)-based technology quantifying 1034 proteins and covering many biological pathways, in particular immune system processes. Using spiked-in recombinant Interleukin-10 (IL-10) and vascular endothelial growth factor D (VEGF-D) in the NIST SRM 1950 plasma standard, we assessed the linearity, sensitivity, precision, and accuracy of the Olink Reveal assay. The results demonstrated strong linear relationships (*R* ^2^ 0.922–0.953) for both IL-10 and VEGF-D across spiked-in concentrations, confirming the robust technical performance for these two proteins in the Olink Reveal platform. The resulting data contain no sensitive or personally identifiable information and are therefore suitable for use in benchmarking and software development. The data are publicly available in the PRIDE repository with identifier PAD000009.

## Graphical abstract

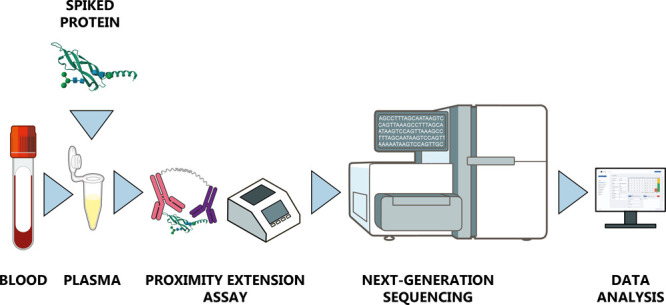

## Introduction

Proteomics is the large-scale study of proteins encompassing their structure, function, interactions, and modifications within biological systems. As it circulates the body, our blood contains proteins expressed by and secreted or leaked from all major organs and tissues. Blood therefore remains an attractive source for the detection of diagnostic and prognostic proteins. For biomarker development and inclusion of proteomics into precision medicine, detailed characterization and quantification of health- and disease-related proteins are required.^1,2^ It is noted, however, that the identification part has received much more attention than the quantitative aspect. Continuing advances in mass spectrometry instrumentation and methodologies^3,4^ and, in particular, increasing competition from affinity-based emerging technologies^5,6^ have led to a resurging interest in plasma proteomics. The latter technologies pursue enhanced speed and robustness of protein biomarker translation and have therefore been designed to allow large-scale population studies, such as those being conducted by UK Biobank.^7,8^

The major technologies used for plasma proteomics have recently been compared by Beimers et al.^9^ and Kirsher et al.,^10^ ranging from depletion and enrichment methods combined with liquid chromatography–tandem mass spectrometry (LC-MS/MS) to several commercially available high-throughput affinity proteomics platforms, such as SomaLogic 7K and 11K, and Olink Explore 3072 and Explore HT (measuring approximately 2900 and 5400 proteins, respectively). The correlation, as measured by median Spearman’s rank correlation coefficient, was found by Kirsher et al. to be high between the two Olink assays (0.85) and between the two SomaLogic products (0.80). Furthermore, both platforms have demonstrated excellent technical repeatability and reproducibility. Nevertheless, moderate concerns remain because of the low correlations between the two affinity proteomics platforms (0.52–0.56), between each affinity platform and the MS methods (0.34–0.68), as well as between different MS platforms (0.36–0.48), and questions are raised about what is actually being measured. However, the primary use case for all technologies has been the detection of differential abundances of proteins between groups of individuals, e.g., patients and healthy controls or different groups of patients. These comparisons are valid due to the previously mentioned excellent assay precision and specificity and do not rely on interplatform correlations or measurement accuracies *per se*.

To validate the analytical performance of any proteomics platform or measurement technology, spike-in experiments can reveal both measurement accuracy and precision as well as linearity and the dynamic range. We here present the first results of two such simple spike-in experiments in the latest Olink assay, Reveal. As with the Explore assays, Olink Reveal is a next-generation sequencing (NGS)-based multiplex proteomics assay that leverages the proximity extension assay technology to relatively quantify a curated panel of protein biomarkers from limited sample input. In the assay, paired antibodies tagged with unique DNA oligonucleotides bind target proteins and, upon proximity, generate DNA barcodes that can be amplified with polymerase chain reaction and quantified by using deep NGS. The curated protein library in the Olink Reveal of 1034 robustly detectable targets was specifically selected to interrogate a majority of biological pathways as curated by Reactome (1780 of 2782 pathways) and is enriched with a selection of 537 immune response-annotated proteins (covering 96% of immune response pathways). This targeted selection facilitates broad profiling of disease-relevant processes in population-scale proteomics studies. A second motivation for these experiments was to provide bioinformaticians and software developers in the proteomics community with some minimally interesting and ground-truth public data from this new platform.

## Methods

### Human Plasma

As a background, we used a single, not previously opened or thawed, vial of the National Institute of Standards and Technology (NIST) SRM 1950 frozen human plasma standard^11^ purchased from Sigma-Aldrich (St. Louis, Missouri, United States). The SRM 1950 is a well-characterized, pooled reference material derived from plasma of 50 male and 50 female healthy donors aged 40–50 years and matching the ethnic distribution of the United States at the time (77 white, 12 African American or black, 4 Asian, 2 American Indian/Alaskan Native, and 5 “other”). While the standard was developed in collaboration between NIST and the National Institutes of Health (NIH) to serve as a quality control benchmark in clinical and analytical applications for the measurement of metabolites, lipids, vitamins, and fatty acids,^12,13^ it also provides a consistent, available, and suitable material for proteomics method validation in proteomics.^14^

### Spike-In Proteins

To explore the linearity, sensitivity, precision, and accuracy of the assay, we spiked the NIST SRM 1950 with Interleukin-10 (IL-10) and vascular endothelial growth factor D (VEGF-D), both recombinantly expressed in HEK 293 cells and purified to ≥95% purity (Sigma-Aldrich, Product Numbers H7541 and SRP3185, respectively). As the actual concentrations of IL-10 and VEGF-D in the NIST plasma were unknown, we used estimates from literature (5.0 pg/mL for IL-10 ^15–17^ and 972 pg/mL for VEGF-D^18^) to calculate the concentrations required for standard additions of 20, 100, and 500% relative to baseline for respective protein in the SRM 1950. For each standard addition, 2.0 μL of recombinant protein diluted in Milli-Q water was added to 98 μL of thawed plasma aliquoted into 1.5 mL Eppendorf tubes, with 2.0 μL Milli-Q water added to 98 μL thawed plasma as a control. The seven tubes spiked with 1.0, 5.0, and 25 pg/mL IL-10; 194.5, 972, and 4860 pg/mL VEGF-D; and the control were then refrozen and kept at −80 °C until the Olink experiment.

### Olink Reveal and Plate Design

For the Olink Reveal experiment, the samples were thawed, and the manufacturer’s protocol was followed (Olink, Uppsala, Sweden). Five technical replicates of each sample were block-randomized and distributed over two 96-well microtiter plates combined in two sequencing runs together with multiple samples from other studies, with 12 samples in the first plate/sequencing run and 23 in the second, with each sample occurring at least once in each plate and five samples occurring twice also in the first plate. The NGS was performed using a NovaSeq X Plus (Illumina, San Diego, California, United States) 1.5B flow cell to sequence each Reveal library with a custom sequencing recipe according to the protocol, generating at least 1B reads per library. The design of the study, including the identity and levels of the spiked-in proteins, were blinded to the technicians performing the assay and the NGS.

### Data Analysis

The sample manifests and count files derived from the raw sequencing reads using an ngs2counts script were combined in NPXMap version 1.0.2 (Olink), producing a single NPX file from both plates and sequencing runs using default settings. The samples not included in this study, but present in the NGS data, were removed in the sample manifests before creating the NPX file. The exported NPX file therefore contains no information, data or metadata, about the other samples, except which proteins were included in the assay (as defined by the Reveal design). The raw NPX file has been deposited to the ProteomeXchange Consortium^19^ via the PRIDE partner repository with the dataset identifier PAD000009. The NPX files were analyzed using an R script available on https://osf.io/as4gv, producing the figures in this paper. Parquet format files and extended format data are also available in the OSF repository.

## Results and Discussion

All samples on both plates passed the manufacturer’s quality control. Figure 1 shows the raw count and NPX response as a function of IL-10 and VEGF-D concentration.

**Figure 1:**
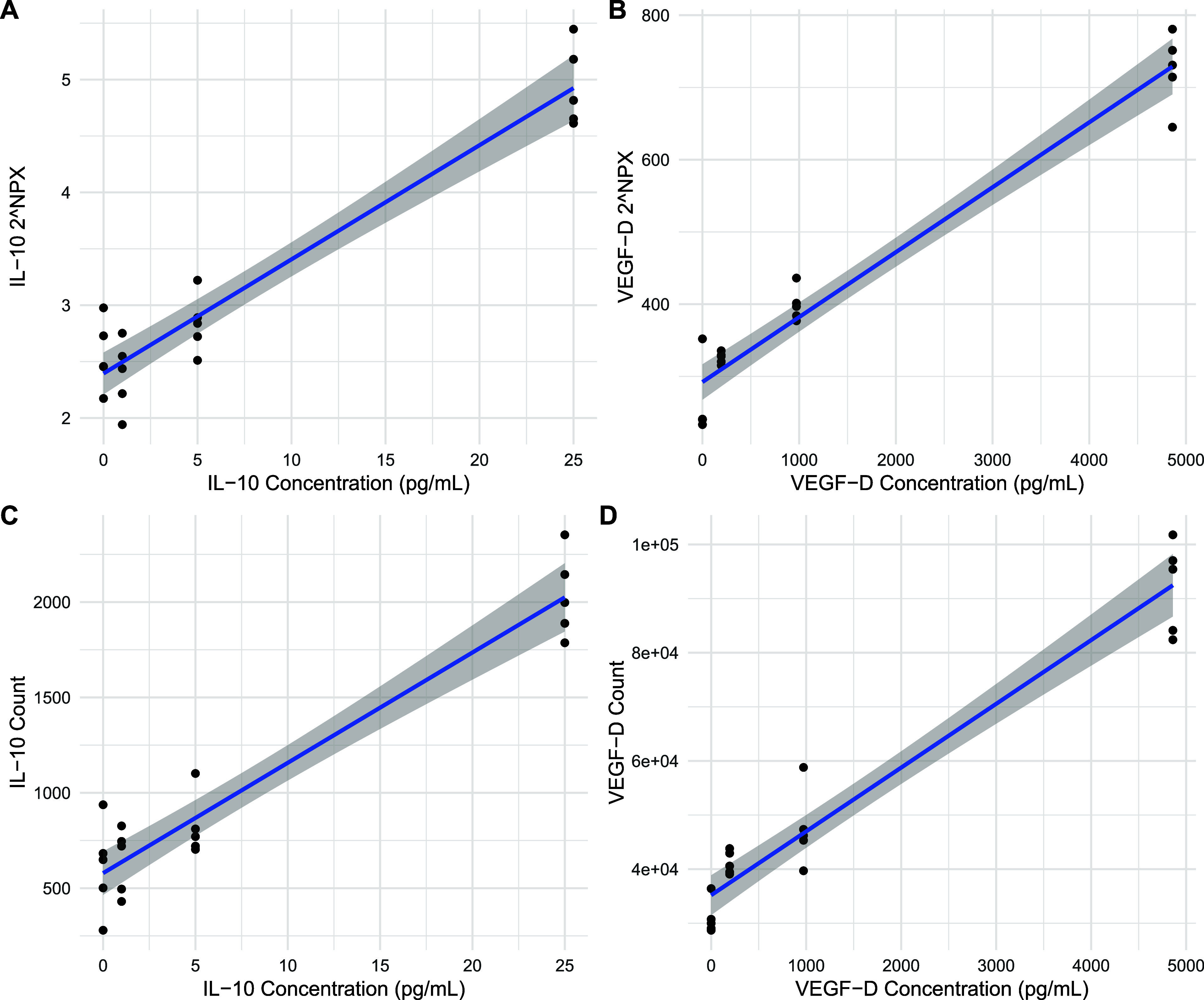
NPX values (log2 and median normalized) for IL-10 (A) and VEGF-D (B), and raw read counts for IL-10 (C) and VEGF-D (D). Coefficient of determination (*R* ^2^) values were higher for VEGF-D than IL-10, and higher for NPX (0.922 for IL-10, 0.953 for VEGF-D) than raw counts (0.910 and 0.939 for IL-10 and VEGF-D, respectively).

Extrapolating from the standard additions, we get 10.0 ± 3.0 pg/mL for IL-10 and 2986 ± 577 pg/mL for VEGF-D. If removing the highest VEGF-D concentration (for example, if it would be outside the linear dynamic range), the extrapolated concentration of 2176 ± 876 pg/mL in the NIST plasma is still considerably higher than the literature estimate. We did not find any significant difference between the 10 and 11 pg/mL IL-10, although the protein was clearly above the limit of detection when compared with the number of reads or NPX values in the negative control (0.25 ± 0.17 vs −1.10 ± 0.17) and the raw counts were also, on average, higher in the spiked samples (1269 ± 242 reads vs 658 ± 128 reads). No significant problems were encountered during sample preparation or analysis, despite this being the first time the assay was performed in our laboratory.

We chose the two recombinant proteins and their spike-in levels based on their expected concentration in the NIST standard and their known variability across diseases. IL-10 is an anti-inflammatory cytokine produced by regulatory T cells, monocytes, and macrophages^20^ that suppress excessive inflammation by inhibiting IL-6, IL-12, and TNF-α (all in the Reveal panel), and downregulating dendritic cell antigen presentation. Reported cohort plasma levels (immunoassay-based) include 3.3 pg/mL^16^ in a study on schizophrenia, 5.0 pg/mL^17^ in a study on epilepsy, and 6.0 pg/mL^15^ in a study on tuberculosis. The protein is not listed as detected in the blood by the Human Protein Atlas. In a multiplexed immunoassay for 190 proteins, the average values were found at 6.6–7.5 pg/mL.^21^

VEGF-D regulates angiogenesis and lymphangiogenesis by binding VEGF R2 and R3.^22^ VEGF-D also plays a critical role in tissue repair and, in cancer, metastasis via lymphatic spread. Its expression is driven by hypoxia and inflammatory mediators, such as IL-1β, IFN-γ (both in the Reveal panel), and TNF-α, making it a potential biomarker and drug target in oncology and vascular diseases.^23^ Average immunoassay cohort/population-based plasma levels have been reported in a study on thyroid cancer at 230 pg/mL,^24^ lymphangioleiomyomatosis at 410 pg/mL,^25^ breast cancer at 970 pg/mL,^18^ and in a study on kidney disease at 1500 pg/mL.^26^ In the mass spectrometry-based peptide atlas,^27^ an average value of 300 pg/mL is reported.

IL-10 was also chosen because of a 1 pg/mL increase (20% at the expected baseline of 5 pg/mL) to challenge the assay. While we did not find any detectable difference in NPX values at these concentrations, differences of this magnitude may still be detectable in larger cohorts. The lowest VEGF-D spike of 972 pg/mL or around 6.5% higher than the actual background was detectable (Figure 1D). The variations in concentration that have been observed in past studies suggest that the assay is fit-for-purpose, at least for these two proteins, in population studies, with all proteins included in the assay having passed the same criteria for analytical performance and robustness.

As the list of 1034 proteins was curated not only based on reagent quality and assay robustness but also by the pathways covered, there is a heavy bias toward proteins involved in immune system processes. As an aside, this also makes it essential to use only the included proteins as the background in any subsequent gene ontology or pathway enrichment analyses, where an enrichment against all genes and proteins will inevitably show an enrichment of immune processes. An additional cautionary comment concerns preanalytical variations. In a recent study, the effects of hemolysis and freeze–thaw cycles on assay results were demonstrated, emphasizing the importance of standardized specimen collection and storage.^28^

## Conclusions

While the results presented here are not revolutionary or even particularly surprising, they constitute the first publicly available data from this new affinity proteomics platform. This, we believe, is in itself of use to anyone writing, managing, or adapting software importing Olink proteomics data (or metadata) for analysis, visualization, or integration with other types of (prote)­omics data. This was indeed the reason for performing these experiments using a commercially available pooled plasma material as the background in the first place.
